# Analysis of the surgical strategy and postoperative clinical effect of thoracic ossification of ligament flavum with dural ossification

**DOI:** 10.3389/fsurg.2022.1036253

**Published:** 2022-10-12

**Authors:** Tao Liu, Sidong Yang, Shuo Tian, Zhen Liu, Wenyuan Ding, Zheng Wang, Dalong Yang

**Affiliations:** Department of Spinal Surgery, The Third Hospital of Hebei Medical University, Shijiazhuang, China

**Keywords:** single-Segment, ligamentum flavum ossification, dural ossification, posterior laminar decompression and internal fixation, postoperative clinical efficacy

## Abstract

**Purpose:**

Our research was designed to analyse the postoperative clinical results of patients suffering from single-segment thoracic ossification of the ligamentum flavum (TOLF) combined with dural ossification (DO) who underwent posterior laminar decompression and internal fixation.

**Methods:**

This retrospective research included thirty-two patients who underwent surgery for ossifying the ligamentum flavum in the thoracic spine between January 2016 and January 2020. Patients were fallen into one group included patients with evidence of DO during surgery, and the other group included patients without evidence of DO. We assessed and compared general clinical characteristics and health-related outcomes before surgery and during follow-up.

**Results:**

The DO group had a longer operation duration, more blood loss, and longer hospital stay (operation time: 94.75 ± 6.78 min vs. 80.00 ± 10.13 min, *p* < 0.001; blood loss: 331.67 ± 50.06 ml vs. 253.00 ± 48.24 ml, *p* < 0.001; length of hospital stay: 13.83 ± 2.76 days vs. 10.05 ± 2.33 days, *p* < 0.001).

**Complications:**

There were 12 cases of cerebrospinal fluid leakage and 1 case of superficial wound infection in the DO group. However, the neurological recovery and health-associated quality of life (HRQOL) scores showed no statistically significant changes between the DO and non-DO groups (*p* > 0.05).

**Conclusions:**

Posterior laminectomy and internal fixation combined with intraoperative resection of the ossified ligamentum flavum and dura is an efficient and relatively safe method for treating TOLF with DO, which can provide satisfactory results. Moreover, DO had no significant effect on postoperative neurological recovery and health-related quality of life scores.

## Introduction

As a chronic degenerative disease of the thoracic spine, ossification of thoracic ligamentum flavum (TOLF) is characterized by heterotropic thoracic OLF (1). In thoracic myelopathy, the incidence is often lower than ossification of the posterior longitudinal ligament (OPLL) and higher than herniation of the nucleus pulposus (HNP) (2). Patients with ossification of the thoracic ligament flavum are more common among East Asian populations, and the onset stage is mainly T9-T12. The prevalence of TOLF varies from 12.0% to 37.7% in different studies (3–5). To date, the pathogenesis of TOLF is not fully understood, and its main factors may be due to osteogenic cytokines (e.g., BMP) and mechanical stress (6–9).

For asymptomatic TOLF patients, surgery is rarely performed; however, thoracic spinal stenosis and spinal cord compression brought by TOLF are usually progressive and difficult to treat conservatively, requiring surgical treatment as soon as possible (10–14). When pressed, the ossified ligamentum flavum is in close contact with the dura mater, which may also ossify. The ossified ligamentum flavum and the dura fuse to form a hard-to-separate bone mass (9), which will undoubtedly enhance the difficulty of operation and the risk of complications such as spinal cord injury, CSF leakage and infection (15–17). Dural tearing of OLF is often considered to be caused by dural adhesion (DA) and dural ossification (DO) (16). Therefore, it is important to choose a more appropriate, safe and effective surgical approach. At the same time, there are relatively few studies on OLF with DO, and often the subjects with OLF are often multisegmental and the number of focal segments is not uniform (15, 16, 18, 19). In order to avoid the selective bias and reduce the error. So this paper includes patients with single-segment thoracic OLF with DO to further add to the analysis of the correlation between the surgical approach to TOLF with DO and postoperative clinical outcomes.

## Materials and methods

### Patient population

The institutional review board of our institution approved the current retrospective research, and a waiver of consent was acquired. The study evaluated patients receiving posterior lamina decompression and internal fixation for single-segment TOLF at our hospital between January 2016 and January 2020. Inclusion criteria: (1) TOLF diagnosed by CT or MRI with a single segmental lesion; (2) complete imaging and clinical data; and (3) patients followed up ≥24 months after surgery. Exclusion criteria: (1) history of thoracic or lumbar surgery; (2) previous history of infection, trauma, tumour, or congenital malformation; and (3) incomplete clinical records. Thirty-two patients (15 women and 17 men) were enrolled in the study. Patients were divided into two groups: the first group included 12 patients (9 females and 3 males) with intraoperative evidence of DO; the second group included 20 patients (6 females and 14 males) without evidence of DO.

### Radiography

Preoperative radiology included general x-rays, computed tomography (CT), and magnetic resonance imaging (MRI). Preoperative plain x-ray exerted a significant effect on deciding the intraoperative site of TOLF. The location and extent of ossified spinal lesions were confirmed by performing CT scans. The location and number of segments influenced by TOLF, spinal cord engagement and any coexisting spinal disorders were determined by performing MRI.

### Surgical procedure

All 32 patients were operated on by the same surgeon. Both groups underwent posterior laminar decompression and internal fixation. The related segment was preliminarily determined based on clinical manifestations and imaging examinations. Under general anaesthesia with endotracheal intubation, the patient was put in the prone position, and the surgical segment was positioned by fluoroscopy before the operation. The median incision on the posterior side was made with the lesion in the centre; the skin, subcutaneous tissue, and thoracolumbar dorsal fascia were incised in sequence; dissection of the paraspinal muscles occurred along the bilateral subperiosteal of the spinous process of the focal segment, stripped to the bilateral articular processes. Then, the spinous process and lamina were spread on both sides with a single hook to expose the intervertebral space of the focal segment. After the surgical incision was exposed and the decompression range was confirmed to be correct, the pedicle of the lesion segment was expanded to make a screw canal, the rongeur bit the spinous process of the corresponding vertebral body, the lamina and ligamentum flavum were removed with a drill, and the OLF in the lesion segment compressed the dura mater. A nerve peeler was used to assess the degree of adhesion of the OLF to the dura. In case of no adhesion, the OLF and lamina were pulled apart with forceps until the head and tail of the ossified tissue opened. One-third of the facet joints were opened outwards bilaterally, exposing the bilateral dura, which could be fully expanded. If there was dural ossification adhesion, the ossified dura mater was excised with a sharp knife, and the dural defect was repaired. Small dural tears were repaired with 4–0 silk sutures, and large dural defects were generally repaired with muscle flaps or adipose tissue. The dura mater was routinely covered with 1–2 layers of gelatine sponge. After thorough decompression, pedicle screws were placed on both sides of the lesion segment, connecting rods were placed on both sides, and nuts were fixed. The decompressed area was reflush with normal saline. A drainage tube was placed next to the median incision; instruments were counted; and the wound was sutured. According to the intraoperative exploration, there were 12 cases in the DO group and 20 in the non-DO group.

All patients were given routine antibiotic therapy within 3 days after surgery. Seven days after the surgery, the patients wore the brace to walk, and brace protection was kept for about 3 months.

### Clinical evaluation

The following data of each patient were recorded: age, sex, BMI, smoking history, alcohol consumption history, history of hypertension, history of diabetes, duration of preoperative symptoms, estimated intraoperative blood loss (EBL), duration of surgery, length of hospital stay (LOH), compression segment of OLF, postoperative complications, the 36-item Short-Form Health Survey (SF-36) (20) and the modified Japanese Orthopaedic Association (mJOA) scoring system were adopted to assess the neurological improvement at preoperative and the last postoperative follow-up, with the highest mark of 11 indicating normal function, a total mark ≤3 indicating severe neurological impairment, 4–6 indicating moderate function, and ≥7 indicating mild function (12). The recovery rate (RR) was calculated as follows: (postoperative JOA score−preoperative JOA score)/(11− preoperative JOA score)× 100 (%), with excellent (RR ≥ 75%), good (75% > RR ≥ 50%), fair (50% > RR ≥ 25%), or poor (RR < 25%) (21).

### Statistical analysis

The measurement data is shown as the mean ± standard deviation, and the counting data are totals and percentages. SPSS software (version 26.0; SPSS, Chicago, Illinois) was adopted to perform all analyses. The comparison of independent variables between the two groups was made by paired sample T test, independent sample T test, *χ*^2^ test or Fisher's exact test, and Mann–Whitney U test. Multivariate logistic regression was used to analyse the factors associated with dural ossification in patients with single-segment thoracic OLF. Modified odds ratios (aORs) and 95% confidence intervals (CIs) were used. A *p* value of <0.05 was of statistical significance.

## Results

### Patient population

**Figure 1:**
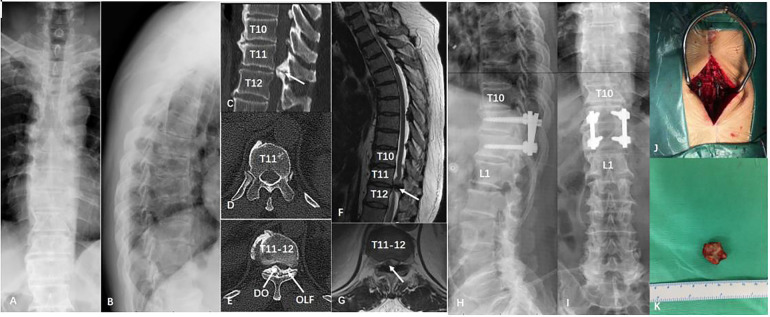
A patient with thoracic ossification of ligamentum flavum in T11/12. (**A,B**) Preoperative x-ray; (**C-E**) Preoperative coronal and axial section computed tomography scan; (**F,G**) Preoperative sagittal and axial section magnetic resonance imaging scan; (**H,I**) Postoperative x-ray; (**J,K**) Intraoperative photo and postoperative sample of ligamentum flavum ossification.

Thirty-two patients with single-level TOLF who underwent posterior lamina decompression and fusion and internal fixation were selected for the current study. Based on the intraoperative exploration, the patients were fallen into the DO group (*n* = 12) and the non-DO group (*n* = 20). Table 1 summarizes the features of these patients. There was no great diversity between the two groups in age, sex, BMI, number of smokers, number of drinkers, incidence of hypertension or diabetes. A typical case is shown in Figure 1.

**Table 1:** Patient Backgrounds.

	OLF with DO	OLF without DO	*p*-Value
No. of patients	12 (37.5%)	20 (62.5%)	
Age (year)	59.25 ± 9.97	57.20 ± 8.19	0.532
Sex			0.055
Male	3	12	
Female	9	8	
BMI (Kg/m^2^)	26.42 ± 3.06	26.85 ± 3.72	0.736
Smoker (*n*)	1 (3.1%)	4 (12.5%)	0.626
Drinking (*n*)	1 (3.1%)	4 (12.5%)	0.626
Hypertension (*n*)	7 (21.9%)	6 (18.8%)	0.150
DM (*n*)	2 (6.2%)	1 (3.1%)	0.540

OLF, ossification of the ligament flavum; DO, dural ossification; BMI, body mass index; DM, diabetes mellitus.

### Clinical characteristics

The mean preoperative symptom duration was 14.92 months in the DO group and 12.10 months in the non-DO group (*p* = 0.527). Compared with the non-DO group, the DO group had a longer operation duration, more blood loss, and longer hospital stay (operation time: 94.75 ± 6.78 min vs. 80.00 ± 10.13 min, *p* < 0.001; estimated blood loss: 331.67 ± 50.06 ml vs. 253.00 ± 48.24 ml, *p* < 0.001; length of stay: 13.83 ± 2.76 days vs. 10.05 ± 2.33 days, *p* < 0.001). Complications included cerebrospinal fluid leakage (DO group: 12, non-DO group: 0), spinal cord injury (DO group: 0, non-DO group: 0), superficial infection (DO group: 1, non-DO group: 0), and screw loosening/failure (DO group: 0, non-DO group: 0) (Table 2).

**Table 2:** Clinical characteristics.

	OLF with DO	OLF without DO	*p*-Value
Preoperative duration of symptoms (months)	14.92 ± 16.05	12.10 ± 13.79	0.527
Operation time (minutes)	89.17 ± 7.33	80.00 ± 10.13	<0.001^a^
LOH (days)	13.83 ± 2.76	10.05 ± 2.33	<0.001^a^
EBL (ml)	331.67 ± 50.06	253.00 ± 48.24	<0.001^a^
No. of complications (*n*)			
Leakage of cerebrospinal fluid	12	0	
Spinal cord injury	0	0	
Superficial infection	1	0	
Screw looseness/failure	0	0	

OLF, ossification of the ligament flavum; DO*,* dural ossification; IQR, interquartile range; LOH, length of hospitalization at postoperative; EBL, estimate blood loss.

^a^ Statistically signifcant.

### Distribution

**Figure 2:**
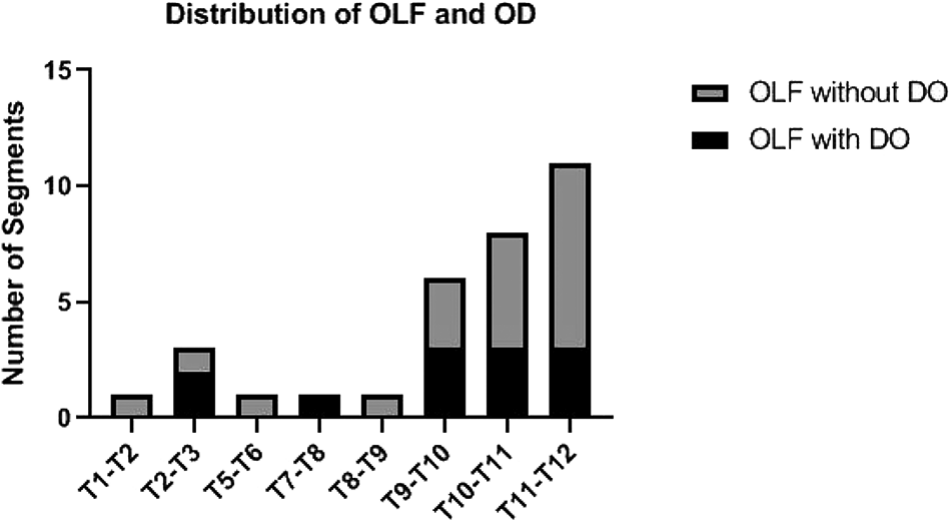
Distribution of OLF and DO: OLF was more common in the lower thoracic spine. More than half (75%) of the DO was located in T9-T12. DO, dural ossification; OLF, ossification of ligamentum flavum.

Figure 2 shows the distribution of TOLF with DO. TOLF is more common in the lower thoracic vertebrae, with more than half (75%) of DO located in T9–T12.

### Postoperative neurological recovery and health-related quality of life scores

Both the DO and non-DO groups showed significant improvements in most health-related outcomes (Table 3). The mean mJOA for all patients gradually improved from 4.67 to 7.75 in the DO group and from 5.75 to 8.40 in the non-DO group. The RR was 50.83 ± 11.09% in the DO group and 53.15 ± 11.29% in the non-DO group. Although the mJOA score of the DO group was lower than that of the non-DO group, no great diversity was observed in neurological function recovery between the group with and without DO (*p* > 0.05). Surgical outcome: The DO group was excellent in 1 (8.3%) patient, good in 6 (50.0%) patients, fair in 5 (41.7%) patients and poor in 0 (0%) patients. The non-DO group was excellent in 1 (5.0%) patient, good in 12 (60.0%) patients, fair in 7 (35.0%) patients and poor in 0 (0%) patients (Table 2). Patients showed no worsened neurological symptoms. No great diversity was found in surgical effect between the two groups (*p* = 0.860). Preoperative spinal cord severity: in the DO group, 1 case was ≥7 (8.3%), 8 cases were 4–6 (66.7%), and 3 cases were ≤3 (25.0%). The non-DO group was divided into 5 patients (18.8%) with ≥7 scores, 14 patients (68.8%) with 4–6 scores, and 1 patient (12.5%) with ≤3 scores. No great diversity was observed in preoperative spinal cord severity between the group with and without DO (*p* = 0.211).

**Table 3:** Comparison of postoperative neurological recovery and health related quality of life.

	OLF with DO	OLF without DO	*p*-Value
mJOA (score)			
Pre	4.67 ± 1.37	5.75 ± 1.55	0.055
F/U	7.75 ± 1.29	8.40 ± 1.23	0.166
Pre VS. F/U	<0.001^a^	<0.001^a^	
RR%	50.83 ± 11.09%	53.15 ± 11.29%	0.576
RR% classification (*n*)			0.860
Excellent	1	1	
Good	6	12	
Fair	5	7	
Poor	0	0	
Preoperative severity of myelopathy (*n*)			0.211
≥7	1	5	
4–6	8	14	
≤3	3	1	
SF-36			
Physical functioning			
Pre	50.83 ± 16.21	61.25 ± 17.53	0.105
F/U	74.58 ± 11.37	81.50 ± 10.77	0.095
Pre vs. F/U	<0.001^a^	<0.001^a^	
Social functioning			
Pre	46.08 ± 17.20	55.85 ± 18.33	0.146
F/U	69.00 ± 14.72	77.15 ± 12.39	0.103
Pre vs. F/U	<0.001^a^	<0.001^a^	
Bodily pain			
Pre	72.83 ± 7.16	73.50 ± 6.19	0.783
F/U	73.67 ± 7.33	75.10 ± 5.67	0.540
Pre vs. F/U	0.137	0.080	
Vitality			
Pre	45.42 ± 8.65	49.00 ± 8.37	0.256
F/U	60.83 ± 6.34	62.25 ± 5.50	0.510
Pre vs. F/U	<0.001^a^	<0.001^a^	
Mental health			
Pre	59.33 ± 8.32	58.45 ± 7.46	0.758
F/U	75.00 ± 6.12	73.80 ± 7.40	0.640
Pre vs. F/U	<0.001^a^	<0.001^a^	
General health			
Pre	50.83 ± 7.33	53.25 ± 7.83	0.394
F/U	68.33 ± 7.49	71.00 ± 6.61	0.301
Pre vs. F/U	<0.001^a^	<0.001^a^	

OLF, ossification of the ligament flavum; DO, dural ossification; mJOA, the modified Japanese Orthopaedic Association; RR, recovery rate; Pre, preoperative; F/U, follow up; SF-36, Short Form-36.

^a^ Statistically signifcant.

These clinical SF-36 outcomes showed no great diversities between the two groups during follow-up. For the SF-36, most measures, including social functioning, physical functioning, mental health, vitality, and general health, were significantly enhanced compared to presurgery, with the exception of bodily pain in the DO group (*p* > 0.05) (Table 3).

### Multivariate logistic regression analysis

The variables related to DO in univariate analysis were operation time, length of hospital stay, and estimated blood loss. According to multivariate logistic regression analysis, the length of hospital stay and estimated blood loss were independently correlated with the DO group (length of hospital stay OR = 2.201, *p* = 0.024, 95% CI 1.110–4.365; estimated blood loss: OR = 1.033, *p* = 0.048, 95% CI 1.000–1.067) (Table 4).

**Table 4:** Multivariate logistic regression analysis of OLF with DO.

Parameters	aOR	95%CI	*p*-Value
Operation time (minutes)	1.124	0.897–1.048	0.309
LOH (days)	1.033	1.000–1.067	0.048^a^
EBL (ml)	2.201	1.110–4.365	0.024^a^

aOR, adjusted odds ratio; CI, confdence interval; LOH, length of hospitalization at postoperative; EBL, estimate blood loss.

^a^ Statistically signifcant.

### Postoperative complications

The ossified dura and ligamentum flavum were directly resected in 12 patients in the DO group, so the dura was torn during the operation, resulting in CSF leakage. Small dural lacerations were repaired with 4-0 silk sutures, while large dural defects were usually repaired with muscle flaps or adipose tissue. CSF leakage was stopped after delayed drainage tube removal and conservative treatment with local pressure was applied for 5∼7 days. One patient developed a superficial wound infection, which was cured after 1∼2 weeks of specific antibiotic treatment.

## Discussion

### Distribution and incidence of TOLF and DO

Patients with TOLF are more common among East Asian populations, and the onset stage is mainly T9–T12 (3–5). This research describes the surgical experience of 32 Chinese patients who underwent single-segment TOLF with or without DO. TOLF was found to be mostly in the lower thoracic spine, with more than half (75%) of the DO located in T9–T12, consistent with previous studies.

The exact incidence of dural ossification in thoracic ligamentum flavum ossification is unclear because most articles mainly describe multisegmental TOLF, few studies have been conducted to explain the combination of DO in single-segment TOLF alone, and it has been suggested that the occurrence of DO is rare. In contrast, among the 32 patients with segmental TOLF included in this paper, 12 patients had combined DO, the prevalence of which was 37.5%, which is like the outcomes reported by Muthukumar and Li et al (16, 18). The incidence of TOLF with DO is relatively high. However, studies on the distribution and prevalence of DO are inadequate, and the present study further provides an additional explanation.

### Surgical procedure and results

Surgical decompression has been the best treatment option for compressive myelopathy because TOLF-associated myelopathy influences the posterior part of the spinal canal (11–13). However, surgical decompression for TOLF with DO has been treated in different ways. Sun et al (11). reported two surgical approaches for the treatment of TOLF combined with DO: dural opening and removal of ossification and floating of the ossified dura by drilling and thinning. Wang et al (12). compared posterior decompression laminectomy with or without internal fixation and fusion therapy, and both surgical methods are effective methods for the treatment of TOLF and can provide satisfactory clinical improvement. In patients with thoracic spinal myelopathy combined with specific types of TOLF, the use of percutaneous total endoscopic posterior decompression (PEPD) is feasible as the most minimally invasive spinal decompression procedure. However, this surgical approach makes it difficult to treat TOLF patients with DO (13). In combination with the surgical approach of the abovementioned studies, this study adopts posterior laminar decompression and internal fixation, and if DO is found intraoperatively, it is removed together with TOLF. The great advantage of this surgical approach is complete decompression and avoidance of ossification recurrence. Although the thoracic spine has restricted motion and better stability compared to the cervical and lumbar spine, our previous study on the clinical efficacy analysis of laminectomy alone and with instrumentation in treating TOLF showed better clinical outcomes and lower perioperative complication rates after internal fixation laminectomy (LI) compared to postoperative laminectomy alone (LA) (22). For insurance purposes, we performed internal fusion of the operated segments to increase stability and safety and reduce the risk of complications in the thoracic spine.

In this study, no diversity was found in the preoperative duration of symptoms between the two groups compared with those without DO, but the DO group had longer surgery, more bleeding, and longer hospital stays. Multivariate logistic regression analysis showed no great diversity in operative time between the two groups, while intraoperative blood loss and length of hospital stay were related to the DO group. If the OLF adhered to the DO during the operation, it would be difficult to separate, so it would need to be removed together; when removing the DO, the surgeon needs to be careful to avoid spinal cord injury because the removal of the DO will cause CSF leakage, so the amount of blood loss during the operation is greater. Postoperative treatment with local pressure and delayed drainage tube removal is needed, so the length of stay is also longer.

In our study, the postoperative recovery of the two groups was mainly good and fair. This is similar to the results of Wang et al (12)., who reported that 8 (24.2%) patients had excellent recovery, 22 (66.7%) patients recovered well, 2 (6%) patients recovered fairly and 1 (3%) patient recovered poorly. However, compared with the complications in other studies, the complications in this study were relatively simple. The main complication in our study was CSF leakage, which was related to the surgical method adopted in this study. During the operation, we found that patients with DO would be directly excised together with TOLF, so patients with DO would suffer from CSF leakage caused by dural defects. Recovery is usually possible with intraoperative repair of the defective dura and with conservative postoperative treatment.

### Postoperative neurological recovery and health-related quality of life scores

There are relatively few reports on the postoperative neurological recovery and quality of life of single segment TOLF combined with DO. Aizawa et al (23). reported that poor recovery after TOLF may be related to inadequate decompression. Sun et al (15). displayed that despite a diversity in JOA scores between the two groups both preoperatively and postoperatively, with the DO group being lower than the non-DO group, there is no statistically significant diversity in neurological recovery between the two groups. In this study, there was no significant difference in postoperative neurological function recovery between the DO and non-DO groups. This may be related to adequate decompression found in both groups. Therefore, under sufficient decompression, DO was not associated with the recovery of neurological function after TOLF. All patients in our research underwent posterior laminar decompression and internal fixation. At the follow-up examination, a significant improvement was found in the preoperative and postoperative JOA scores. However, most of the recovery was incomplete, with a mean value of 50.83 ± 11.09% for the DO group and 53.15 ± 11.29% for the non-DO group for RR. Similar to previous reports (14, 24, 25).

No great diversity was found in HRQOL between the two groups during follow-up. Nevertheless, most postoperative indicators of patients, including social function, physical function, mental health, vitality and overall health, were significantly enhanced, but the improvement in postoperative bodily pain showed no difference between the group with or without DO. This may be because DO mainly compresses the spinal cord centrally rather than the nerve roots, and therefore, the improvement in somatic pain is not significant.

There were some limitations in this study. First, the duration of follow-up was short, and longer follow-up is therefore needed to verify the outcomes of this research. Second, small sample size may affect the statistical results. Third, there may be some inherent biases in the retrospective study design and patient data. However, this article examines patients with single-segment TOLF with DO to add a more nuanced perspective on this type of disease.

## Conclusions

Posterior laminectomy and internal fixation combined with intraoperative resection of the ossified ligamentum flavum and dura is an efficient and relatively safe method for treating TOLF with DO, which can provide satisfactory results. Moreover, DO had no significant effect on postoperative neurological recovery and health-related quality of life scores.

## Data Availability

The original contributions presented in the study are included in the article/Supplementary Material, further inquiries can be directed to the corresponding author/s.
